# Impact of mechanical ventilation on clinical outcomes in ICU-admitted Alzheimer’s disease patients: a retrospective cohort study

**DOI:** 10.3389/fpubh.2024.1368508

**Published:** 2024-03-27

**Authors:** Han Liu, Qun Liang, Yang Yang, Min Liu, Boyang Zheng, Shilin Sun

**Affiliations:** ^1^Institute for Global Health, University College London, London, United Kingdom; ^2^First Affiliated Hospital of Heilongjiang University of Chinese Medicine, Harbin, China; ^3^The Second Affiliated Hospital of Guangzhou Medical University, Guangzhou, China; ^4^Heilongjiang University of Chinese Medicine, Harbin, China

**Keywords:** Alzheimer’s disease, ICU, mechanical ventilation, management strategies, mortality, MIMIC-IV

## Abstract

**Background:**

Alzheimer’s disease (AD) is increasingly recognized as a pressing global public health issue, demanding urgent development of scientific AD management strategies. In recent years, the proportion of AD patients in Intensive Care Units (ICU) has been on the rise. Simultaneously, the use of mechanical ventilation (MV) is becoming more prevalent among this specific patient group. Considering the pathophysiological characteristics of AD, the application of MV in AD patients may lead to different outcomes. However, due to insufficient research data, the significant impact of MV on the prognosis of AD patients in the ICU remains unclear. Therefore, we conducted this study to comprehensively evaluate the potential influence of MV on the survival rate of AD patients in the ICU.

**Methods:**

We obtained data from the MIMIC-IV database for patients diagnosed with AD. Using propensity score matching (PSM), we paired patients who received MV treatment with those who did not receive treatment. Next, we conducted Cox regression analysis to evaluate the association between MV and in-hospital mortality, 7-day mortality, 28-day mortality, 90-day mortality, 4-year mortality, length of hospital stay, and ICU stay.

**Results:**

The data analysis involved a cohort of 641 AD patients spanning from 2008 to 2019, inclusive. Following a 1:2 propensity score matching (PSM) procedure, 300 patients were successfully paired, comprising 123 individuals who underwent MV treatment and 177 who did not. MV demonstrated an association with an elevated risk of in-hospital mortality (HR 5.782; 95% CI 2.981–11.216; *p* < 0.001), 7-day mortality (HR 6.353; 95% CI 3.014–13.392; *p* < 0.001), 28-day mortality (HR 3.210; 95% CI 1.977–5.210; *p* < 0.001), 90-day mortality (HR 2.334; 95% CI 1.537–3.544; *p* < 0.001), and 4-year mortality (HR 1.861; 95% CI 1.370–2.527; *p* < 0.001). Furthermore, it was associated with a prolonged length of ICU stay [3.6(2.2,5.8) vs. 2.2(1.6,3.7); *p* = 0.001]. In the subgroup analysis, we further confirmed the robustness of the results obtained from the overall population. Additionally, we observed a significant interaction (*p*-interaction <0.05) between age, admission type, aspirin use, statin use, and the use of MV.

**Conclusion:**

In patients with AD who are receiving treatment in the ICU, the use of MV has been linked to higher short-term, medium-term, and long-term mortality rates, as well as prolong ICU stays. Therefore, it is crucial to break away from conventional thinking and meticulously consider both the medical condition and personal preferences of these vulnerable patients. Personalized treatment decisions, comprehensive communication between healthcare providers and patients, formulation of comprehensive treatment plans, and a focus on collaboration between the ICU and community organizations become imperative.

## 1 Introduction

Alzheimer’s disease (AD) is the most prevalent neurodegenerative disorder, estimated to encompass 60–70% of all dementia cases worldwide (1, 2). Currently, over 30 million people globally are affected by AD. Without breakthroughs in medical advancements for the prevention, deceleration, or cure of AD, it is projected that by 2050, the affected population may soar to approximately 101 million. Consequently, AD has become a mounting global public health concern, resulting in substantial economic losses (3–5). Surpassing even cancer, AD now holds the top position as the most worrisome disease in the United States, with an estimated 6.7 million Americans aged 65 and above currently grappling with the condition (6). Numerous endeavors have been undertaken to devise disease-modifying approaches; however, the intricate pathophysiology of AD has thus far thwarted the discovery of interventions capable of significantly slowing or preventing its clinical progression (1, 7). In this context, seeking scientific strategies for AD management might represent another crucial avenue to address the current challenges posed by AD.

An increasing number of scholars recognize the importance of AD management strategies. They advocate for clinicians to establish proactive and flexible personalized approaches, providing compassionate care for individuals and caregivers (8–12). However, there is currently relatively little focus on management strategies for AD patients in the ICU. With the global aging population intensifying, there is a noticeable rise in the admission rates of older adults AD patients to the ICU. Over the past 20 years, their demand for ICU services has more than doubled, concurrently leading to higher post-ICU admission mortality rates (13, 14). Given these trends, delving into the management strategies for AD patients in the ICU becomes particularly crucial. Ventilation strategy is an indispensable component of ICU management, with mechanical ventilation (MV) playing a pivotal role. Each year, as many as 20 million individuals require admission to the ICU and undergoing MV, aiming to enhance their short-term survival rates (15–17). There is an increasing body of evidence suggesting a gradual rise in the application of MV in AD patients (12, 18). However, AD patients exhibit specific differences in clinical manifestations and physiology, potentially resulting in divergent management strategies compared to non-AD patients (5, 19). For instance, certain studies have revealed that short-term MV contributes to the pathological features of AD. It leads to an elevated accumulation of the amyloid-ß_1-40_ (Aß_1-40_) peptide and neuroinflammation in the cerebral region. Surprisingly, this is accompanied by a reduction in blood–brain barrier permeability (20–22). Therefore, more research data are needed to support the use of MV in AD patients admitted to the ICU. Nevertheless, due to a lack of research data, the question of whether MV significantly benefits AD patients or influences their survival remains unanswered.

Hence, we conducted this study to assess the impact of MV on the survival rate of AD patients admitted to the ICU. By concentrating on this specific cohort, our aim is to offer robust data support to improve the understanding and management of the medical needs unique to this patient population.

## 2 Methods

We collected data from the MIMIC-IV database v2.2 using Navicat Premium v16.1.7, with a particular focus on patients with AD who either received or did not receive MV. The establishment of the MIMIC-IV database follows the best practices in scientific computing, representing a genuine and openly accessible resource. This database encompasses data from over 73,000 patients treated in the ICU at Beth Israel Deaconess Medical Center from 2008 to 2019. It includes essential demographic information, laboratory results, ICU monitoring records, and treatment prescriptions. The latest version of MIMIC-IV has been officially released on the Physionet website.^1^ Our co-author, Shilin Sun, obtained authorized access to use this database (Authorization Number: 12281929). This study involves the analysis of an existing, anonymized public database and has received approval from the Institutional Review Board (IRB) at the Massachusetts Institute of Technology (MIT) and Beth Israel Deaconess Medical Center (BIDMC). Therefore, it is exempt from the requirement for informed consent (23, 24). In this study, all reporting followed the guidelines of the Strengthening the Reporting of Observational Studies in Epidemiology (STROBE) criteria (25).

### 2.1 Study population

Given that the MIMIC-IV database spans from 2008 to 2009, a period during which the United States was transitioning from the International Classification of Diseases, 9th edition (ICD-9) to the 10th edition (ICD-10), our retrospective study simultaneously included patients who met the AD criteria within both ICD-9 and ICD-10. Specifically, we identified AD patients based on the standards of ICD-9 (3310) and ICD-10 (G30, G300, G301, G308, and G309) (26–28).

Exclusion criteria were as follows: (1) patients under the age of 18; (2) patients with multiple ICU admissions were only included in the analysis for their first ICU admission (29); (3) patients who were discharged or died within 24 h after ICU admission; (4) patients with missing potential risk variables for death.

### 2.2 Mechanical ventilation use

The researchers assessed the use of mechanical ventilation by retrieving data for each patient within the first 24 h of ICU admission from the MIMIC-IV database.

### 2.3 Covariates

We included a set of covariates to evaluate the prognosis of AD patients admitted to the ICU, including heart rate, mean arterial pressure (MAP), respiratory rate, SPO2, glucose levels, hematocrit levels, hemoglobin levels, platelet count, white blood cell (WBC) count, creatinine levels, simplified acute physiology score (SAPS) II score, mv modes, and comorbid conditions such as myocardial infarction, congestive heart failure, peripheral vascular disease, cerebrovascular disease, chronic pulmonary disease, mild liver disease, renal disease, use of statins, and use of aspirin (14, 30). Additionally, important information from hospital admission records, including demographic characteristics, marital status, insurance details, and admission type, was also included. These variables account for various aspects of patient health-related behaviors that could introduce potential confounding effects in patients receiving MV therapy.

### 2.4 Outcome

The primary outcome of this study is in-hospital mortality, and secondary outcomes encompass mortality rates at 7 days, 28 days, 90 days, and 4 years, along with the lengths of hospital and ICU stays.

### 2.5 Statistical analysis

Continuous variables with a normal distribution were reported as mean ± standard deviation and compared between groups using independent-samples *t*-test. Meanwhile, continuous variables with a skewed distribution were presented as median (IQR) and compared between groups using the Mann–Whitney *U* test. Categorical variables were described as numbers and percentages, and between-group comparisons were performed using the chi-squared test or Fisher’s exact test as appropriate.

Propensity Score Matching (PSM) is an extremely useful matching technique that intuitively achieves balance in the treatment group, thereby reducing bias and assessing the impact of treatment effects on outcomes. The PSM method has its advantages and limitations. By balancing the relationship of confounding factors between the treatment and control groups, PSM achieves a more objective analysis. However, it is important to note that PSM only allows for the adjustment of measurable confounding factors, and all multivariate adjustment methods are subject to this limitation (31). Due to the non-randomized nature of this study and considering significant differences in baseline characteristics, we PSM in our study using a greedy nearest neighbor matching with a caliper of 0.1 standard deviations of the logit of the estimated propensity score (32). Patients were matched at a 1:2 ratio, so each patient who received MV during ICU hospitalization was paired with two patients who did not undergo MV treatment. We calculated the standardized mean difference (SMD) to assess the effectiveness of PSM in reducing differences between the two groups (33).

The Cox model is a regression technique used for survival analysis in epidemiological and clinical research. The model is based on estimating the hazard ratio (HR) associated with specific risk factors or predictor variables for a given endpoint, serving as a fundamental statistical method for addressing etiological and prognostic hypotheses in the field of epidemiology and clinical research (34). We used a multivariate Cox regression model to adjust for confounding variables selected based on univariate analysis with a *p*-value <0.05 and potential confounders identified by our team’s clinical expertise. This was undertaken to assess the association between the use of MV and the outcome of mortality (35).

In the subgroup analysis, we conducted an initial interaction analysis. Investigating the results of the interaction analysis, we explored whether the relationship between MV management and in-hospital mortality is influenced by factors such as gender, age, admission type, race, marital status, insurance, comorbidities, and medication use. Furthermore, we delved into the exploration of specific subpopulations.

Statistical analyses were conducted using IBM SPSS Statistics version 26.0 and R 4.2.2 software. A *p*-value of <0.05 was considered statistically significant.

## 3 Results

### 3.1 Population

**Figure 1:**
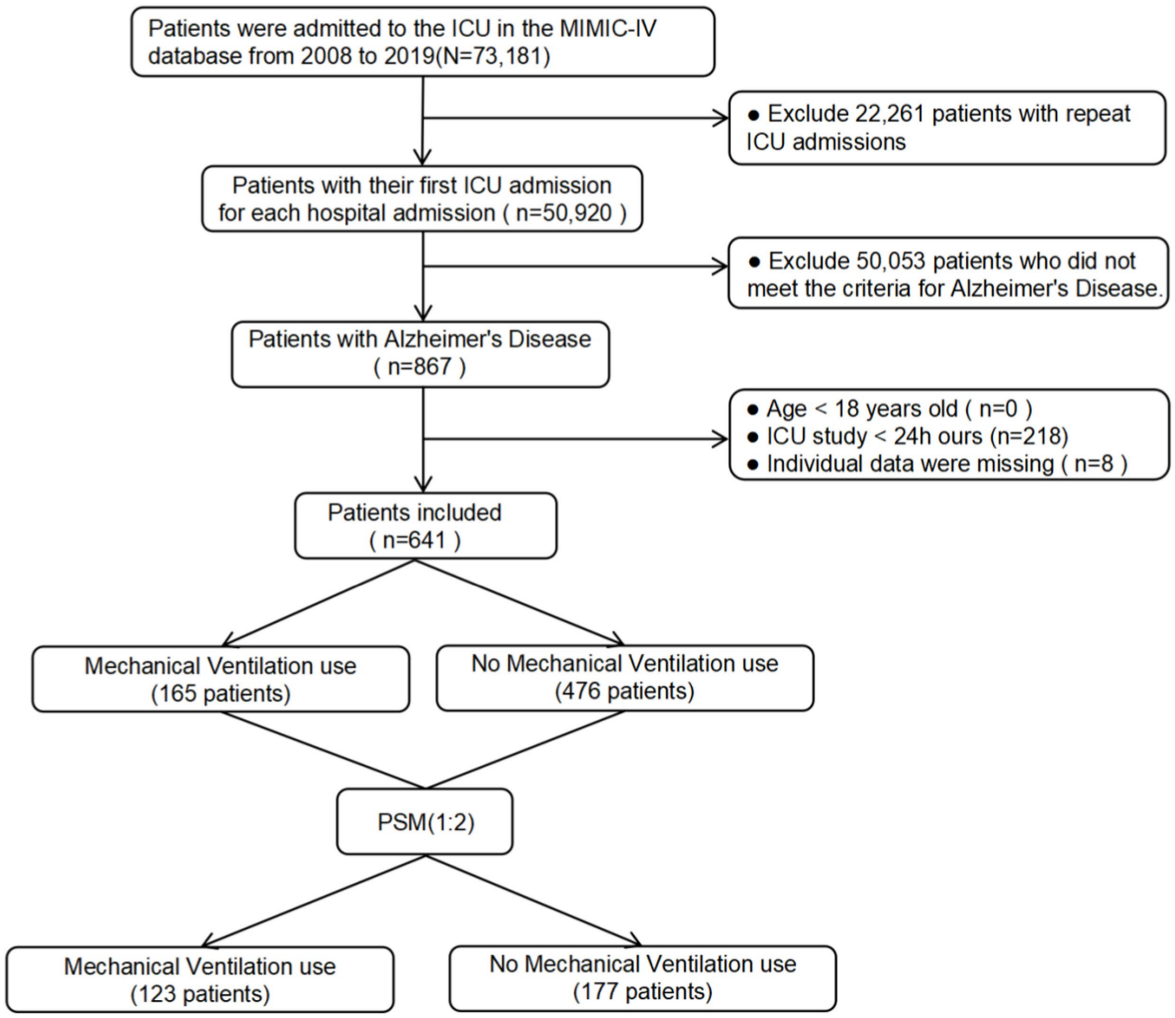
The flow chart of the study.

A total of 867 patients diagnosed with AD were identified based on the ICD-9 and ICD-10 standards. After applying the exclusion criteria, 641 eligible patients were included in the study. Among these patients, 165 cases (25.7%) underwent MV during their ICU stay. A visual representation of the patient selection process is provided in Figure 1.

As depicted in Table 1, there are notable differences in several baseline characteristics between the two patient groups in the original cohort, including age, sex, race, MAP, respiratory rate, SPO_2_, glucose levels, WBC, SAPS II score, and medication use. Within the MV group, there were significant reductions in MAP and respiratory rate, while variables such as SPO_2_, glucose levels, WBC, and SAPS II score showed a significant increase.

**Table 1:** Baseline characteristics between groups before and after PSM.

Variables	Before matching				After matching			
	No mechanical ventilation use group	mechanical ventilation use group	*p*-value^1^	SMD^2^	No mechanical ventilation use group	mechanical ventilation use group	*p*-value^1^	SMD^2^
*n*	476	165			177	123		
Age (%)			0.017				0.54	
>65	454 (95.4)	149 (90.3)		−0.172	162 (91.5)	110 (89.4)		−0.027
≤65	22 (4.6)	16 (9.7)		0.172	15 (8.5)	13 (10.6)		0.027
Sex, female (%)	280 (58.8)	82 (49.7)	0.042	−0.183	93 (52.5)	66 (53.7)	0.849	0.065
Admission type (%)			0.355				0.406	
Emergency	404 (84.9)	135 (81.8)		−0.079	149 (84.2)	99 (80.5)		−0.063
Elective	72 (15.1)	30 (18.2)		0.079	28 (15.8)	24 (19.5)		0.063
Ethnicity, white (%)	348 (73.1)	99 (60.0)	0.002	−0.268	114 (64.4)	80 (65.0)	0.91	0.108
Marital status (%)			0.965				0.921	
Married	182 (38.2)	65 (39.4)		0.024	65 (36.7)	48 (39.0)		0.025
Single/divorced	114 (23.9)	39 (23.6)		−0.007	48 (27.1)	32 (26.0)		−0.019
Other	180 (37.8)	61 (37.0)		−0.018	64 (36.2)	43 (35.0)		−0.008
Insurance (%)			0.191				0.66	
Medicare	350 (73.5)	123 (74.5)		0.023	136 (76.8)	89 (72.4)		−0.075
Medicaid	5 (1.1)	5 (3.0)		0.115	3 (1.7)	2 (1.6)		−0.024
Other	121 (25.4)	37 (22.4)		−0.072	38 (21.5)	32 (26.0)		0.088
Heart rate (bpm)	83 ± 17	83 ± 16	0.706	0.035	83 ± 16	82 ± 15	0.69	−0.023
MAP (mmHg)	82 ± 10	79 ± 9	0.002	−0.292	80 ± 10	80 ± 10	0.458	−0.014
Respiratory rate (bpm), [median (IQR)]	19.3 (17.2, 22.7)	17.9 (16.2, 20.5)	<0.001	−0.404	18.1 (16.4, 21.0)	17.9 (16.2, 20.5)	0.699	0.024
SPO_2_ (%), [median (IQR)]	96.66 (95.38, 97.79)	98.55 (97.25, 99.15)	<0.001	0.638	97.52 (96.28, 98.63)	98.28 (97.04, 98.93)	0.003	0.024
Glucose (mg/dL), [median (IQR)]	129 (107, 161)	136 (119, 164)	0.007	0.166	135 (111, 167)	135 (120, 161)	0.395	−0.06
Hematocrit (%)	33.5 ± 5.9	33.6 ± 6.2	0.894	0.012	33.5 ± 6.1	33.7 ± 6.0	0.716	−0.01
Hemoglobin (g/L)	10.92 ± 1.99	10.95 ± 2.00	0.87	0.015	10.87 ± 2.09	10.95 ± 1.97	0.748	0.002
Platelet (x 10^12^), [median (IQR)]	201 (157, 262)	194 (151, 240)	0.163	−0.216	191 (151, 260)	196 (153, 246)	0.853	−0.059
WBC (x10^9^), [median (IQR)]	10.3 (7.9, 13.8)	11.3 (9.1, 15.0)	0.021	0.127	10.4 (8.0, 14.4)	11.2 (9.0, 14.0)	0.283	0.063
SCr (mg/dL), [median (IQR)]	1.05 (0.80, 1.50)	1.05 (0.80, 1.40)	0.336	−0.136	1.10 (0.85, 1.55)	1.05 (0.80, 1.38)	0.145	−0.105
SAPS II score, [median (IQR)]	39 (34, 47)	44 (37, 54)	<0.001	0.402	41 (34, 51)	43 (36, 52)	0.21	−0.016
Comorbidity disease (%)								
Myocardial infarct	87 (18.3)	28 (17.0)	0.706	−0.035	30 (16.9)	20 (16.3)	0.875	0.011
Congestive heart failure	142 (29.8)	56 (33.9)	0.325	0.087	57 (32.2)	35 (28.5)	0.489	−0.06
Peripheral vascular disease	57 (12.0)	23 (13.9)	0.511	0.057	23 (13.0)	15 (12.2)	0.838	0.023
Cerebrovascular disease	96 (20.2)	41 (24.8)	0.206	0.108	43 (24.3)	32 (26.0)	0.735	0.028
Chronic pulmonary disease	84 (17.6)	40 (24.2)	0.065	0.154	36 (20.3)	24 (19.5)	0.86	−0.028
Mild liver disease	15 (3.2)	4 (2.4)	0.793	−0.047	8 (4.5)	3 (2.4)	0.534	−0.106
Renal disease	110 (23.1)	37 (22.4)	0.857	−0.016	42 (23.7)	26 (21.1)	0.598	−0.039
Medications use (%)								
Aspirin	101 (21.2)	59 (35.8)	<0.001	0.303	44 (24.9)	33 (26.8)	0.701	0.034
Statin	125 (26.3)	64 (38.8)	0.002	0.257	51 (28.8)	40 (32.5)	0.492	0.05
MV mode								
CMV/ASSIST/AutoFlow	-	70(42.4)			-	53(43.1)		
CMV/ASSIST	-	38(23.0)			-	27(22.0)		
APV (cmv)	-	33(20.0)			-	24(19.5)		
PSV/SBT/SPONT/Ambient	-	15(9.1)			-	13(10.6)		
Other	-	9(5.5)			-	6(4.9)		

^1^Pearson’s Chi-squared test; Fisher’s exact test; Welch Two Sample *t*-test; Wilcoxon rank sum test. ^2^Standardized mean difference. CMV, controlled mechanical ventilation; ASSIST, assist-control ventilation; APV, assist pressure ventilation; PSV, pressure support ventilation; SBT, spontaneous breathing trial; SPONT, spontaneous.

**Figure 2:**
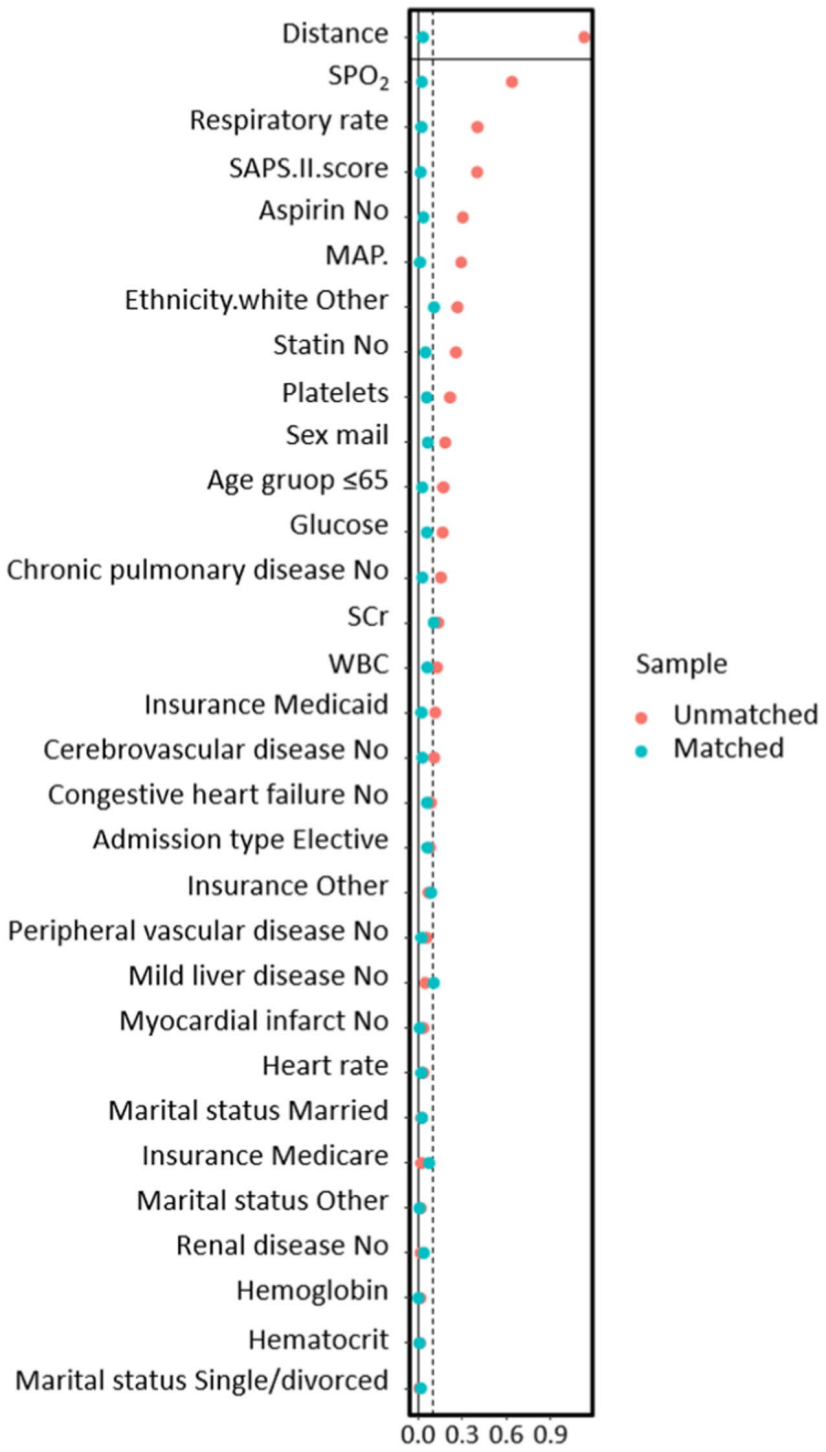
The relationship between SMD and all covariates before and after propensity score matching models.

In the PSM analysis, 300 patients were successfully paired, consisting of 123 individuals who underwent MV treatment and 177 who did not. Following matching, there was good balance in baseline characteristics between the two groups, with all variables having a SMD of less than 10% (Figure 2).

### 3.2 Association between utilization of mechanical ventilation and clinical outcomes

In the initial cohort, we observed a significant association between the use of MV and a higher in-hospital mortality rate (HR 2.865; 95% CI 1.890–4.345; *p* < 0.001). After adjusting for potential confounding factors, the association between the use of MV and higher in-hospital mortality remained statistically significant (HR 4.581; 95% CI 2.806–7.479; *p* < 0.001). We also assessed the impact of MV on 7-day, 28-day, 90-day, and 4-year mortality rates, as well as the length of hospital stay and ICU stay. The results indicated that the use of MV was associated with a higher 7-day mortality rate (HR 5.211; 95% CI 2.959–9.176; *p* < 0.001), higher 28-day mortality rate (HR 2.511; 95% CI 1.708–3.693; *p* < 0.001), higher 90-day mortality rate (HR 1.564; 95% CI 1.114–2.196; *p* = 0.01), higher 4-year mortality rate (HR 1.319; 95% CI 1.038–1.675; *p* = 0.023), prolonged hospital stay [8.7 (4.6, 14.2) vs. 6.8 (4.1, 10.7), *p* = 0.007], and extended ICU stay [3.3 (2.1, 6.0) vs. 2.1 (1.5, 3.3); *p* < 0.001] (Table 2).

**Table 2:** Association between mechanical ventilation use and clinical outcomes in Alzheimer’s disease patients.

	No MV use	MV use	*p-*value	HR	Lower 95% CI	Upper 95% CI
Pre-matched cohort	*n* = 476	*n* = 165				
Primary outcome						
In-hospital mortality, *n* (%)^a^	47(9.9)	42(25.5)	<0.001	4.581	2.806	7.479
Secondary outcomes						
7-day mortality, *n* (%)^a^	33(6.9)	34(20.6)	<0.001	5.211	2.959	9.176
28-day mortality, *n* (%)^a^	87(18.3)	55(33.3)	<0.001	2.511	1.708	3.693
90-day mortality, *n* (%)^a^	138(29.0)	64(38.8)	0.01	1.564	1.114	2.196
4-year mortality, *n* (%)^a^	262(55.0)	110(66.7)	0.023	1.319	1.038	1.675
Length of hospital stay (day), [median (IQR)]	6.8(4.1,10.7)	8.7(4.6,14.2)	0.007	1.787	0.496	3.078
Length of ICU stay (day), [median (IQR)]	2.1(1.5,3.3)	3.3(2.1,6.0)	<0.001	1.672	1.008	2.335
Post-matched cohort	*n* = 177	*n* = 123				
Primary outcome						
In-hospital mortality, *n* (%)^a^	12(6.8)	34(27.6)	<0.001	5.782	2.981	11.216
Secondary outcomes						
7-day mortality, *n* (%)^a^	10(5.6)	28(22.8)	<0.001	6.353	3.014	13.392
28-day mortality, *n* (%)^a^	28(15.8)	43(35.0)	<0.001	3.210	1.977	5.210
90-day mortality, *n* (%)^a^	43(24.3)	50(40.7)	<0.001	2.334	1.537	3.544
4-year mortality, *n* (%)^a^	90(50.8)	83(67.5)	<0.001	1.861	1.370	2.527
Length of hospital stay (day), [median (IQR)]	6.9(4.8,11.0)	8.2(4.9,14.0)	0.371			
Length of ICU stay (day), [median (IQR)]	2.2(1.6,3.7)	3.6(2.2,5.8)	0.001	1.295	0.523	2.066

HR, hazard ratio; CI, confidence interval; ICU, intensive care unit; IQR, interquartile range. ^a^Adjusted for all the factors (sex, age, admission type, marital status, insurance, ethnicity, heart rate, MAP, respiratory rate, SPO2, glucose, hematocrit, hemoglobin, platelets, WBC, SCr, myocardial infarct, congestive heart failure, peripheral vascular disease, cerebrovascular disease, chronic pulmonary disease, mild liver disease, renal disease, SAPS II score, mv mode, aspirin use and statin use).

After conducting PSM, we observed similar results in the PSM cohort. The use of MV, adjusted for potential confounding factors, remained associated with higher in-hospital mortality (HR 5.782; 95% CI 2.981–11.216; *p* < 0.001). Additionally, we found that MV was correlated with higher 7-day mortality (HR 6.353; 95% CI 3.014–13.392; *p* < 0.001), higher 28-day mortality (HR 3.210; 95% CI 1.977–5.210; *p* < 0.001), higher 90-day mortality (HR 2.334; 95% CI 1.537–3.544; *p* < 0.001), and higher 4-year mortality (HR 1.861; 95% CI 1.370–2.527; *p* < 0.001). There was also a prolonged ICU stay associated with MV [3.6 (2.2, 5.8) vs. 2.2 (1.6, 3.7); *p* = 0.001] (Table 2).

### 3.3 Subgroup analysis

**Figure 3:**
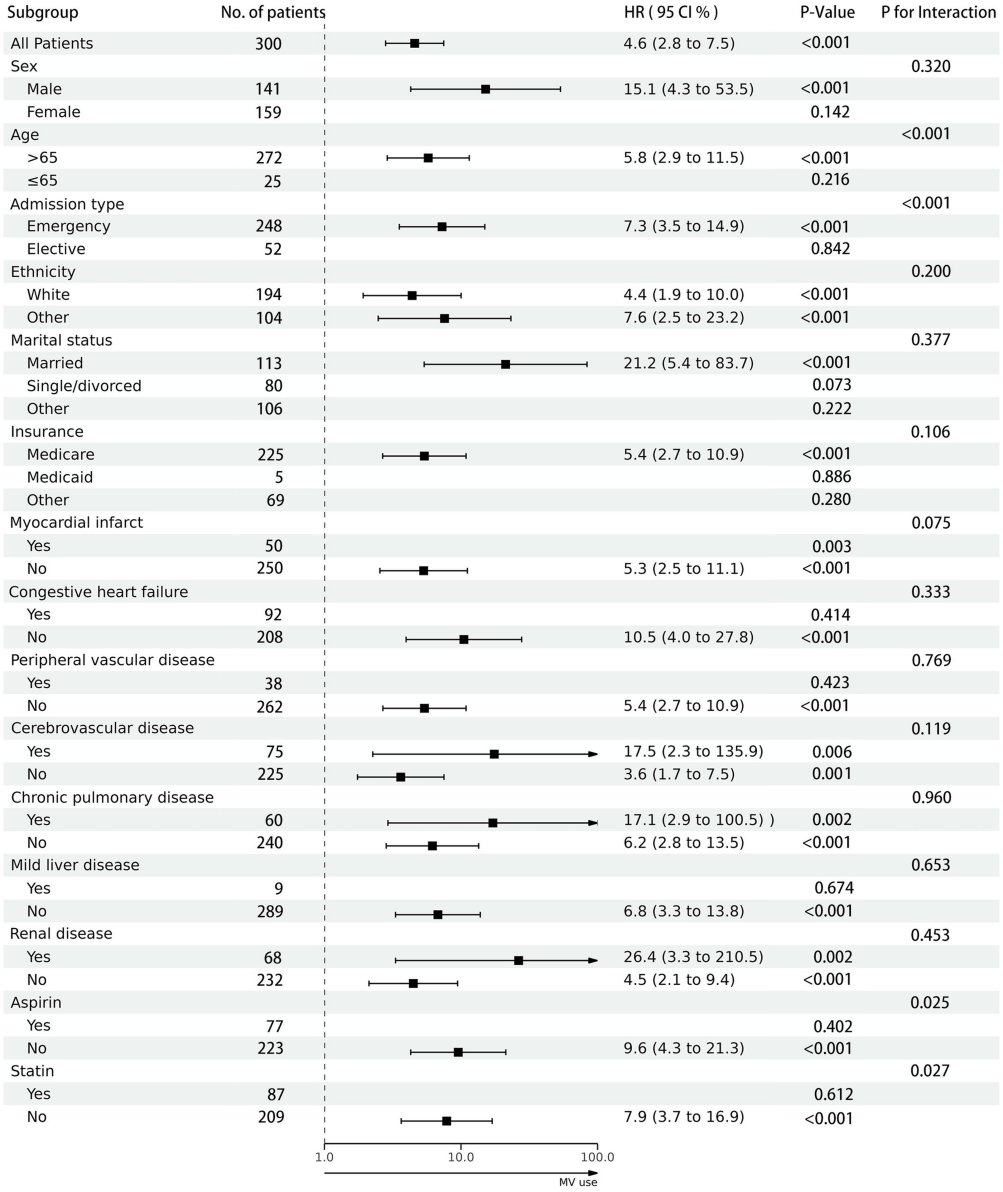
The association between mechanical ventilation use and in-hospital mortality in subgroups. Each stratification adjusted for all the factors (sex, age, admission type, marital status, insurance, ethnicity, heart rate, MAP, respiratory rate, SP02, glucose, hematocrit, hemoglobin, platelets, WBC, SCr, myocardial infarct, congestive heart failure, peripheral vascular disease, cerebrovascular disease, chronic pulmonary disease, mild liver disease, renal disease, SAPS II score, mv mode, aspirin use and statin use).

As shown in Figure 3, within subgroups stratified by factors such as gender, race, marital status, and insurance, or by comorbid conditions including myocardial infarction, congestive heart failure, peripheral vascular disease, cerebrovascular disease, chronic pulmonary disease, mild liver disease, and renal disease, no significant interactions were observed (*P*-interaction >0.05). This suggests that the results obtained from the overall population are dependable, signifying a significant association between the use of MV and higher mortality rates.

Additionally, we observed a significant interaction between age, admission type, aspirin use, and statin use with the use of MV (*P*-interaction <0.05). Through in-depth analysis, we identified specific subpopulations, namely patients aged 65 or younger, those admitted through elective procedures, and those using aspirin or statins, where there was no statistically significant difference in hospital mortality rates associated with the use of MV.

## 4 Discussion

Our research findings indicate a significant correlation between the use of MV in AD patients receiving treatment in the ICU and a higher rate of in-hospital mortality. The cohort results further demonstrate that MV may lead to increased short-term (in-hospital and 7-day), medium-term (28-day and 90-day), and long-term (4-year) mortality rates, as well as prolong ICU stays. This discovery has the potential to challenge conventional ICU management strategies for AD patients, prompting increased attention from scholars on the management approaches within the ICU for this specific patient population. These research findings underscore the necessity for personalized treatment strategies for AD patients, involving crucial considerations in patient care, medical decision-making, and public health. Firstly, the study reveals a significant association between MV and short-term mortality rates in AD patients, emphasizing the need for close monitoring and urgent care during the early stages of MV treatment. Healthcare professionals must remain vigilant to promptly identify and address potential complications or deteriorations that may arise during MV treatment. Additionally, for the ICU team and family members, understanding the increased short-term mortality rates may impact resource allocation and treatment expectations, necessitating more sensitive communication to coordinate treatment goals and provide support and comfort as needed. Secondly, the elevated mortality rates at 28 and 90 days highlight the critical post-ICU discharge period, emphasizing the need for enhanced emphasis on rehabilitation strategies, including regular follow-ups and rehabilitation plans, to promote both physiological and psychological recovery in patients. From the perspective of the healthcare system, the increase in medium-term mortality rates may pose challenges to the planning and allocation of medical resources, potentially requiring additional long-term care resources and rehabilitation services to better meet the needs of this specific patient population. Finally, the emphasis on an increase in long-term (4-year) mortality rates underscores the need for long-term, lifelong management and care for AD patients with chronic illnesses. This may involve regular follow-ups, medication management, and lifelong rehabilitation support. With the impact on long-term mortality rates, medical decision-making becomes more complex. Physicians need to provide comprehensive decision support, including discussions and planning for end-of-life care, to ensure that patients and their families understand and accept the potential outcomes of treatment.

In our subgroup analysis, we further confirmed the robustness of the results obtained from the overall population. Additionally, through in-depth investigation, we found no statistically significant differences in the use of MV and hospital mortality within specific subpopulations. These particular subpopulations include patients aged 65 or below, those admitted through elective procedures, and individuals using aspirin or statin medications. For these particular cohorts, the balance between the risks and benefits of MV may differ from other groups. This suggests that ICU healthcare professionals, when dealing with patients aged 65 or younger or those admitted electively with AD, can flexibly assess the necessity of MV and make decisions based on individual patient circumstances. Furthermore, these findings provide insights indicating that aspirin or statin medications might, to some extent, mitigate the risks associated with MV for AD patients. The potential connection could be related to the regulatory effects of these medications on inflammation, hemodynamics, or other physiological processes. However, specific mechanisms require further in-depth research for clarification. The results of these subgroup analyses also emphasize the importance of personalized decision-making when considering MV treatment. Factors such as age, admission method, and medication use may play a crucial role in determining the effectiveness of MV. This offers more specific information for shared decision-making between doctors and patients, enabling physicians to engage in more informed discussions with patients and collaboratively devise the most suitable treatment plans.

In previous laboratory studies, Lahiri et al. (20) found that short-term MV significantly increased soluble Aß_1-40_ and neuroinflammatory cytokines in the brains of AD mice. Additionally, it markedly reduced blood–brain barrier permeability, thereby promoting the neuropathology of AD. Bilotta’s et al. (21) study suggested that ventilation strategies may influence the central nervous system by altering pulmonary inflammatory responses, indicating a medically induced impact of MV on the brain. Sasannejad et al. (22) research group proposed that MV and acute blood–brain barrier weakening due to systemic inflammation could exacerbate existing chronic blood–brain barrier dysfunction in AD, rendering the brain susceptible to both amyloid-beta accumulation and cytokine-mediated hippocampal damage. In contrast, our research focuses on the clinical outcomes of MV in AD patients undergoing treatment in the ICU, providing tangible clinical evidence to support the findings of these laboratory studies. It serves as a crucial bridge in translating laboratory research outcomes into practical applications. This comprehensive information equips clinical practitioners to thoroughly assess treatment options and offers robust data support for enhancing ICU management strategies for AD patients.

In previous cohort studies, researchers such as Lagu et al. (36) analyzed the use of MV in a national sample of hospitalized patients in the United States (NIS) from 2001 to 2011, distinguishing between those with and without dementia. They predicted a fourfold increase in the rate of hospitalized dementia patients receiving MV. This study underscores the need to facilitate early discussions on care goals for older adults patients with advanced terminal conditions (36). On the other hand, Sullivan et al. (37) conducted a retrospective cohort study using 2016–2017 Medicare data on Medicare Advantage patients with late-stage AD and related dementias. They found an increased proportion of these patients receiving invasive MV during hospitalization, along with higher 30-day and 365-day mortality rates compared to Medicare patients (37). Additionally, semi-structured in-depth interviews conducted by Sun et al. (18) with healthcare professionals revealed that ICU-level care is generally unwelcome for late-stage dementia patients. Furthermore, Zhu et al. (38) study found that community residents with late-stage dementia are more likely to receive life-sustaining treatments at the end of life, which may be inconsistent with their end-of-life wishes. Strengthening advance care planning and doctor-patient communication can improve the quality of end-of-life care for dementia patients. In comparison, our study is focused on AD patients within the ICU, as AD is the most prevalent type of dementia. We conducted a detailed analysis of the impact of MV on the short-term, medium-term, and long-term mortality rates of AD patients, providing ICU healthcare professionals, patients, their families, and other stakeholders with intuitive data. Our goal is to promote a more nuanced and personalized management approach for AD patients and to provide support for well-informed medical decisions.

Currently, an increasing number of scholars recognize the significance of AD management strategies. Some scholars believe that the foundation of current AD treatment involves both pharmacological and non-pharmacological approaches, along with nursing plans. These strategies are based on patient-centered psychological education, shared goal setting, and the robust triadic relationship formed among clinical physicians, and patients with their caregivers (8, 19). Some researchers propose that cognitive and functional assessments, genetic typing, CSF and PET imaging biomarkers, as well as MRI, are crucial tools available for stratifying patients based on AD pathology and clinical staging. This stratification is considered vital in establishing precise and personalized medical care, optimizing disease prevention and drug therapy, thereby slowing or preventing cognitive decline, while minimizing adverse effects (39). Furthermore, some scholars have found that a collaborative management model involving nurses, primary care providers, and community organizations can reduce emergency department visits, shorten hospital stays, increase the utilization of end-of-life care services, and delay long-term care for AD patients (40). In comparison to these studies, our research focuses on the impact of MV on the clinical outcomes of AD patients in the ICU, presenting strategies for managing AD patients in the ICU.

Specifically, based on the results of this study and the current research status of AD, we propose the following management strategies for AD patients in the ICU:

(a) Personalized Treatment Decisions: the findings of this study emphasize the necessity to abandon a one-size-fits-all treatment approach when treating AD patients in the ICU. Healthcare professionals should have a clear understanding of the pathophysiological characteristics of AD. Patient stratification based on clinical disease features, pathological biomarkers, genotype, and demographic risk factors is recommended. A meticulous assessment of each AD patient admitted to the ICU is crucial, especially when considering life-sustaining treatments such as MV. Healthcare providers need to comprehensively consider individual differences, disease severity, and expected treatment outcomes to formulate the most appropriate treatment plan.
(b) Comprehensive Doctor-Patient Communication: prior to implementing life-sustaining treatments like MV, comprehensive communication between healthcare providers and patients is of paramount importance. This includes explaining the potential effects and risks of treatment to patients and their families while respecting the personal values and wishes of the patient. Through effective communication, shared decision-making between healthcare providers and patients can be achieved, ensuring the treatment plan aligns with the patient’s expectations and values.
(C) Development of Comprehensive Treatment Plans: if the use of life-sustaining treatments such as MV is unavoidable, the study recommends developing corresponding short-term, medium-term, and long-term treatment and rehabilitation plans. This comprehensive plan should consider the patient’s overall condition, encompassing physiological, psychological, and social aspects, to maximize treatment effectiveness and minimize adverse effects.
(D) Emphasis on Collaborative Management Models between ICU and Community Organizations: establishing close collaborations with community organizations is crucial in this collaborative management model, which includes public education, nursing guidance, end-of-life care, and other cooperative aspects. Collaboration with community health institutions, nursing teams, and various stakeholders can better support patient recovery and comprehensive management. Public education activities can raise awareness in the community about ICU and AD, helping patient families better understand the importance of treatment decisions and providing an enhanced support network for patients. This collaborative model also holds the potential to provide ongoing care after patients leave the ICU, ensuring they receive comprehensive support.

This study provides valuable insights into the survival rates of AD patients undergoing MV in the ICU. However, it is crucial to acknowledge certain limitations. Firstly, due to the retrospective nature of the study, selection bias is inevitable and may introduce uncontrollable confounding factors, such as patients’ baseline characteristics and health conditions. Despite employing methods like PSM and COX regression analysis to adjust for potential confounding factors, the possibility of residual bias still exists. Secondly, the study’s timeframe only covers the population from 2008 to 2019, potentially failing to capture the long-term course of AD fully. Additionally, the limitations of the MIMIC-IV database, insufficient information on the reasons for mechanical ventilation use, and a lack of details regarding causes of death, along with potential issues related to sensitivity and the absence of severity information when identifying dementia patients using ICD codes, restrict a more in-depth analysis of the indications for mechanical ventilation and mortality outcomes. These limitations may impact the generalizability of our study results. Thirdly, this study solely focuses on the relationship between MV and survival rates in AD patients, without directly comparing the MV sensitivity between AD and non-AD patients. Future research should pay more attention to comparing MV sensitivity between AD and non-AD patients. Furthermore, being a single-center study, future validation is needed through more prospective cohort studies and multicenter trials. Despite these limitations, this study offers crucial information for understanding the impact of MV on the survival rates of AD patients in the ICU. The findings underscore the necessity of personalized treatment decisions, comprehensive doctor-patient communication, development of comprehensive treatment plans, and the importance of collaboration between ICU and community organizations in the treatment of AD patients in the ICU. Future research should continue to explore the impact of different treatment strategies on the long-term prognosis of AD patients in the ICU, providing deeper insights into the specific needs and best practices for this patient population.

## 5 Conclusion

In patients with AD who are receiving treatment in the ICU, the use of MV has been linked to higher short-term, medium-term, and long-term mortality rates, as well as prolong ICU stays. Therefore, it is crucial to break away from conventional thinking and meticulously consider both the medical condition and personal preferences of these vulnerable patients. Personalized treatment decisions, comprehensive communication between healthcare providers and patients, formulation of comprehensive treatment plans, and a focus on collaboration between the ICU and community organizations become imperative.

## Data availability statement

The raw data supporting the conclusions of this article will be made available by the authors, without undue reservation.

## Ethics statement

The studies involving humans were approved by The Beth Israel Deaconess Medical Center ICU. The studies were conducted in accordance with the local legislation and institutional requirements. The participants provided their written informed consent to participate in this study. Written informed consent was obtained from the individual(s) for the publication of any potentially identifiable images or data included in this article.

## Author contributions

HL: Writing – original draft, Writing – review & editing. QL: Writing – original draft, Writing – review & editing. YY: Writing – original draft. ML: Writing – original draft. BZ: Writing – original draft. SS: Writing – original draft, Writing – review & editing.

## Footnotes

^1^https://physionet.org/content/mimiciv/2.2/
